# A Novel *AlN*/*Sc*_0.2_*Al*_0.8_*N*-Based Piezoelectric Composite Thin-Film-Enabled Bioinspired Honeycomb MEMS Hydrophone

**DOI:** 10.3390/mi16040454

**Published:** 2025-04-11

**Authors:** Fansheng Meng, Chaoshuai Zhang, Guojun Zhang, Renxin Wang, Changde He, Yuhua Yang, Jiangong Cui, Wendong Zhang, Licheng Jia

**Affiliations:** Science and Technology on Electronic Test and Measurement Laboratory, North University of China, Taiyuan 030051, China; sz202206139@st.nuc.edu.cn (F.M.); sz202306056@st.nuc.edu.cn (C.Z.); zhangguojun1977@nuc.edu.cn (G.Z.); wangrenxin@nuc.edu.cn (R.W.); hechangde@nuc.edu.cn (C.H.); yangyuhua@nuc.edu.cn (Y.Y.); jgcui@nuc.edu.cn (J.C.); wdzhang@nuc.edu.cn (W.Z.)

**Keywords:** AlN/ScAlN composite film, hydrophone, sensitivity, piezoelectric, equivalent noise density

## Abstract

An innovative design of a hydrophone based on a piezoelectric composite film of $AlN/Sc_{0.2}Al_{0.8}N$ is presented. By designing a non-uniform composite sensitive layer, the dielectric loss and defect density are significantly reduced, while the high-voltage electrical characteristics of scandium-doped aluminum nitride are retained. X-ray diffraction analysis shows that the sensitive films have excellent crystal quality (FWHM is 0.34°). According to the standard underwater acoustic calibration test, the device exhibits full directivity with a minimum deviation of ±0.5 dB at 1 kHz frequency, sound pressure sensitivity of −162.9 dB (re: 1 V/μPa) and equivalent noise density of 46.1 dB (re: 1 μPa/*√*Hz). The experimental results show that the comprehensive performance of the piezoelectric heterostructure hydrophone meets the standard of commercial high-end hydrophones while maintaining mechanical stability, and provides a new solution for underwater acoustic sensing.

## 1. Introduction

As a core component in underwater acoustic detection, hydrophones play an irreplaceable role in marine exploration [1], resource exploration [2], underwater communication [3], and military defense [4]. Their fundamental functionality relies on an acoustic–electric conversion mechanism that transforms underwater acoustic signals into processable electrical signals, enabling the acquisition and transmission of subaqueous information. When acoustic waves propagate through water and interact with hydrophones, the internal sensing elements generate corresponding electrical signals through pressure-induced deformation [5,6].

Currently, the mainstream piezoelectric material system includes polycrystalline materials (such as zinc oxide (ZnO) [7], aluminum nitride (AlN) [8] and lead zirconate titanate (PZT) [9]) and monocrystal materials (such as lithium niobate (LiNbO_3_) [10]) and lead magnesium niobate–lead titanate (PMN-PT) [11]), in which monocrystal materials are popular materials for broadband hydrophone design. Piezoelectric film hydrophones are based on the direct piezoelectric effect. Sound pressure deforms the membrane, causing the internal charge displacement of the material to produce an electric charge proportional to the sound pressure. The change in acoustic pressure drives the synchronous fluctuation of charge, which is converted into voltage signal by the electrode [12], and the acoustoelectric conversion is realized by the piezoelectric equations [13]. The traditional piezoelectric ceramic hydrophone cannot meet the demand of ocean exploration due to the limitation of size and performance [14,15,16,17]. AlN has become the core material of MEMS [18] due to its superior piezoelectric and dielectric properties, but its scandium doping strengthening strategy has reliability defects caused by element segregation [19]. By constructing an AlN/ScAlN heterostructure, this design has been extended to micro-sensors such as resonators [20] and accelerometers [21], providing a new paradigm for the development of high-performance hydrophones, while maintaining high piezoelectric activity while restraining lattice distortion and reducing energy dissipation.

MEMS piezoelectric hydrophone uses a sensor unit array architecture, on the one hand, by improving the array fill factor [22], by an imitation of the honeycomb structure, and using internal and external difference electrodes [23,24] and a tent plate structure, resulting in a significant boost in pressure sensitivity. In addition, hydrophones also play an important role in the fields of underwater acoustic imaging [25], marine environmental monitoring [26], underwater target positioning [27] and ranging systems [28]. With the increasing demand for ocean exploration, the performance requirements for hydrophones are also increasing, which are mainly reflected in three aspects: high sensitivity, low noise density and miniaturization [29,30].

With the growing demand for ocean exploration, high sensitivity, low noise density and miniaturization have emerged as critical performance indicators for hydrophones. In this work, a high-performance piezoelectric composite film hydrophone based on AlN/ScAlN is developed. Compared to conventional piezoelectric ceramic hydrophones, this device achieves remarkable improvements in sensitivity and noise suppression while overcoming the size constraints of traditional devices via miniaturization. This work describes a hydrophone, film design and performance characterization system based on AlN/ScAlN piezoelectric composite film preparation technology. Performance evaluations indicate that the composite thin film structure not only improves mechanical stability and reliability but also maintains a high piezoelectric response by inhibiting Sc segregation, reducing dielectric loss, and minimizing lattice distortion.

These advantages enable it to effectively extract weak signals and adapt to complex underwater environments, especially suitable for high-precision underwater acoustic detection applications, such as deep-sea bioacoustic monitoring underwater communication systems and miniaturized sonar arrays, which provide innovative solutions for marine resource exploration and military defense.

## 2. Hydrophone Design and Simulation

In this work, ScAlN rather than AlN is selected as the piezoelectric sensing layer for the benefit of higher receiver sensitivity, since ScAlN has a softer structure together with higher electronegativity, and hence resulting in a larger piezoelectric constant (ScAlN about −0.71 $C/m^{2}$). Scandium-doped aluminum nitride (ScAlN) can significantly improve the piezoelectric response by substituting Sc atoms for Al sites to induce lattice distortion, but excessive doping will lead to increased dielectric loss, residual stress accumulation and Sc segregation on the surface, resulting in crystal quality degradation and decreased sound transmission performance. Therefore, AlN/ScAlN composite films are designed with a heterogeneous structure, and the synergistic effect of AlN substrate and Sc_0.2_Al_0.8_N surface layer is used to effectively inhibit the precipitation of Sc elements, improve the lattice matching degree, and reduce the X-ray diffraction half-peak width and surface roughness. Doping scandium (Sc) enhances the A-axis lattice constant due to the larger ionic radius of Sc compared to Al, while reducing the c/a ratio, thereby increasing the lattice mismatch between the film and the substrate. The full-width at half-maximum (FWHM) of the X-ray diffraction (XRD) peak rocking curve increases from 1.3° for pure AlN to 2° for 42% Sc-doped samples, indicating that high Sc concentrations lead to crystal orientation dispersion, which is attributed to lattice strain and a reduction in coherence length. Scanning electron microscopy (SEM) analysis reveals that an increase in Sc concentration results in larger grain sizes and the accumulation of abnormal grains, significantly deteriorating the surface roughness [31]. This strategy achieves the cooperative optimization of low defect density and mechanical stability while maintaining high piezoelectricity.

In this work, utilizing the COMSOL Multiphysics 6.2 simulation platform, a systematic multi-physics coupling numerical simulation of piezoelectric composite thin-film hydrophones was conducted. Firstly, the three-dimensional model of the sensing element is established, and the material properties of the multi-layer structure are configured according to the experimental parameters, as shown in Table 1. The geometric model is constructed by Boolean operation and stretch operation. By defining the material constitutive relation layer by layer, the geometric configuration is improved, and then the piezoelectric coupling multi-physical field model is established to realize the bidirectional coupling mechanism between electrostatic field and solid mechanical field. The mesh density with convergence optimization is used to discretize the whole model. An incident pressure field of 1 Pa was applied as the boundary condition at the top of both the water and air domains, while perfectly matched layers (PMLs) were used on all other surfaces to simulate an unbounded domain.

**Figure 1:**
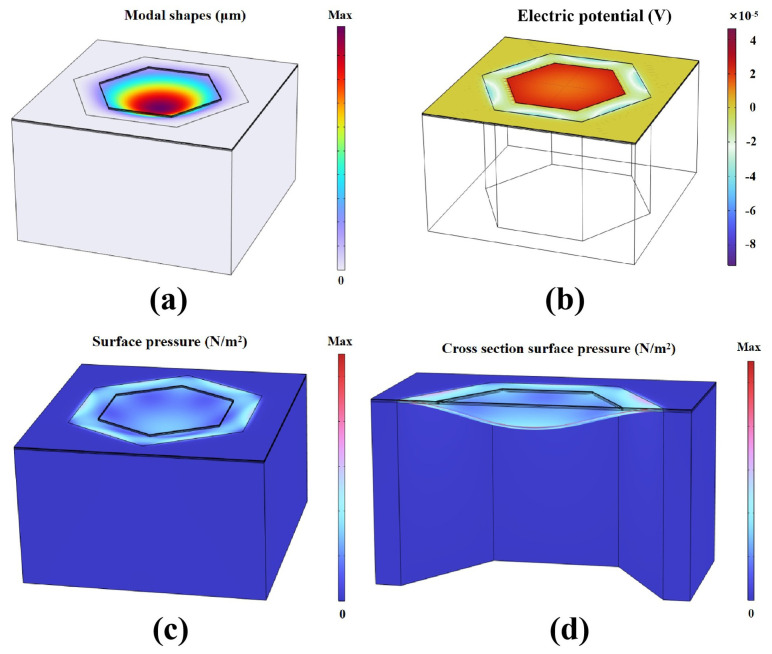
Numerical simulation results of the piezoelectric composite film hydrophone: (**a**) The first-order vibration-mode distribution, comparing the modal deformation and frequency difference; (**b**) three-dimensional potential distribution under electric-mechanical coupling to characterize the potential gradient between electrodes; (**c**) surface stress nephogram per unit sound pressure load; (**d**) cross-sectional dynamic stress distribution, which varies reversibly with the phase of the sound wave.

The first-order modal mode of the hydrophone is obtained by adding the characteristic frequency study (Figure 1a). The numerical results show that the resonant frequencies of the device in air and underwater environments are 130 kHz and 60 kHz, respectively.

In a further study of frequency domain characteristics, the sweep frequency range of 10–1000 Hz is set, and the spatial distribution law of electric potential under acoustic pressure load is extracted by a post-processing algorithm (Figure 1b).

In the electrostatic physical field, by setting the ground state of the bottom electrode, the piezoelectric sensitive layer can conserve charge and the top electrode has suspension potential. Through the study of “frequency domain”, the frequency range is set to 10–1000 Hz, the step size is 10 Hz, and the potential diagram under this frequency band is obtained. The sensitivity expression is thus built using the global calculation as follows:

(1) $$\mathrm{SR}=20\mathrm{lg}(\mathrm{ES}.\mathrm{Fp}1.v_{0}\times 10^{-6})$$

where $ES.Fp1.v_{0}$ is the charge generated by the suspension potential. To draw a one-dimensional plot group, the independent variable is the frequency, and the dependent variable is the sound pressure sensitivity. The result shows that the intrinsic acoustic pressure sensitivity of the sensor without signal gain is −205 dB (re: 1 V/μPa). In the steady-state analysis module, the distribution characteristics of the stress field on the surface of the hydrophone were analyzed by applying unit amplitude sound pressure excitation (Figure 1c). Figure 1d shows the dynamic cross-sectional stress distribution of the piezoelectric composite film under the action of sound wave, in which compressive stress is present in the inner region and tensile stress is present in the outer region. This distribution changes reversibly with the phase of the sound wave (alternating positive and negative pressure), which more directly verifies the high synchronization of the mechanical response characteristics of the piezoelectric film with the phase of the sound wave. Our work further reveals that optimizing the critical dimension of the top electrode to 0.7 times the diameter of the sensing diaphragm enables effective coverage of the sensitive region where charge polarity remains invariant and stress orientation is uniformly maintained, thereby significantly improving the sensor’s reception sensitivity.

The resonant frequency $f_{air}$ of the hydrophone in air and $f_{water}$ in water can be expressed as

(2) $$f_{air}=\frac{\lambda_{0}\times t}{2\pi \times d^{2}}\sqrt{\frac{E_{eq}}{12\times \left(1-\upsilon_{eq}^{2}\right)\times \rho_{eq}}}$$

(3) $$f_{\mathrm{water}}=\frac{f_{\mathrm{air}}}{\sqrt{1+\frac{0.67d\rho_{\mathrm{water}}}{\rho_{\mathrm{eq}}}}}$$

In this context, the characteristic dimension *d* represents the diameter of the outer circle of the hexagonal diaphragm, *t* denotes the film thickness, and $\rho_{water}$ signifies the density of water. $E_{eq}$, $\rho_{eq}$, and $\upsilon_{eq}$ represent the equivalent elastic modulus, equivalent density, and equivalent Poisson’s ratio, respectively. $\lambda_{0}$ is the correction factor.

**Figure 2:**
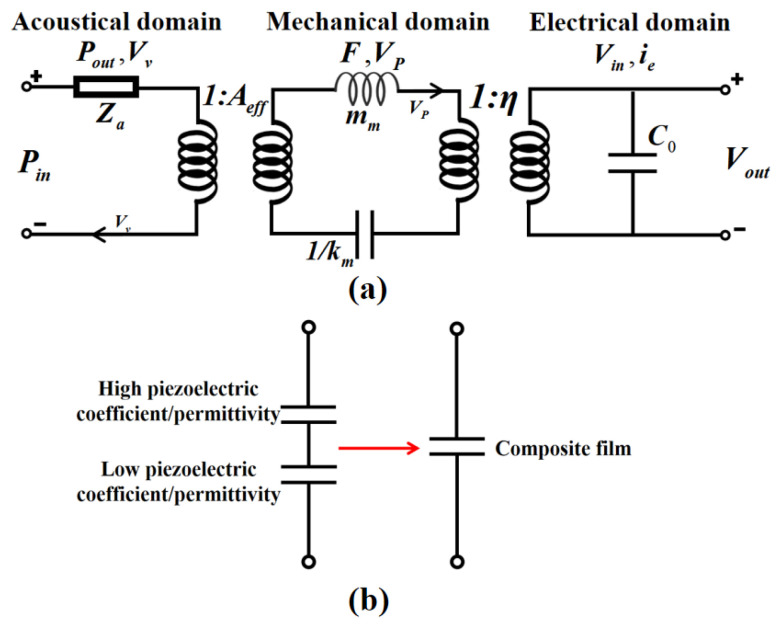
Equivalent circuit model. (**a**) The multi-physical domain coupling model of the sensing unit; acoustic domain features include incident sound pressure, $P_{in}$, and equivalent acoustic impedance, $Z_{a}$; mechanical domain consists of effective mass, $m_{m}$, and effective stiffness, $K_{m}$; electrical domain represents the energy conversion relationship through static capacitance, $C_{0}$, and output terminal voltage, $V_{out}$. (**b**) Theoretical mode for composite film.

As the receiving end, the equivalent circuit model of the sensing unit for the piezoelectric composite film hydrophone is illustrated in Figure 2a.

In the acoustic domain, $P_{in}$ denotes the pressure applied to the surface of the sensing element, while $Z_{a}$ represents the acoustic impedance. Within the mechanical domain, $m_{m}$ signifies the effective mass of the sensing unit, and $k_{m}$ indicates the effective stiffness. In the electrical domain, $C_{0}$ represents the intrinsic capacitance of the device, and $V_{out}$ is the voltage received at the surface of the sensitive unit. $A_{eff}$ is utilized as the equivalent acting area, typically one-third of the surface area of the vibrating diaphragm.

The receiving sensitivity $S_{RX}$ can be expressed by the equivalent circuit model as

(4) $$S_{RX}=\frac{G\cdot A\cdot FA}{\eta }\frac{3\eta^{2}Z_{\mathrm{ele}}}{Z_{tot}+\eta^{2}Z_{\mathrm{ele}}}$$

In the acoustic domain, the acoustic impedance $Z_{a}$ characterizing a clamped radiator is determined as

(5) $$Z_{a}=\frac{\rho_{eq}c}{A_{eff}}\left(jX_{r}+R_{r}\right)$$

where $R_{r}$ and $X_{r}$ are the acoustic terms of resistance and inductance, respectively.

The electromechanical coupling factor η can be expressed as

(6) $$\eta =kd_{s}$$

where $d_{s}$ is the static displacement of the sensitive unit.

In this context, *G* denotes the voltage amplification factor of the pre-amplifier circuit, $Z_{ele}$ corresponds to the electrical impedance of the hydrophone element, $Z_{tot}$ represents the equivalent impedance under coupled mechanical–acoustic field conditions, *A* signifies the effective surface area of hydrophone elements, and FA quantifies the spatial efficiency coefficient of the sensor’s geometric configuration.

The directivity of the piezoelectric composite film hydrophone, expressed as $D_{e}\left(\theta_{i}\right)$, can be expressed as

(7) $$D_{e}\left(\theta_{i}\right)=\frac{48J_{3}\left(ka\sin\theta_{i}\right)}{{\left(ka\sin\theta_{i}\right)}^{3}}$$

where $\theta_{i}$ is the angle from the focus to a single hydrophone array, and α is the equivalent radius of a single array.

The electromechanical coupling factor ($K_{t}^{2}$) of the thickness of the composite film is calculated by the following formula:

(8) $$K^{2}=\frac{e_{31}^{2}}{\varepsilon_{r}\varepsilon_{0}c_{33}}$$

(9) $$K_{t}^{2}=\frac{K^{2}}{1+K^{2}}$$

where $e_{31}$, $\varepsilon_{r}$, $\varepsilon_{0}$, and $c_{33}$ are the piezoelectric constant, relative dielectric constant, vacuum dielectric constant, and stiffness constant of the hydrophone, respectively.

It is worth noting that although scandium-doped aluminum nitride (ScAlN) materials exhibit significantly increased $e_{31}$ values, a synchronous increase in their dielectric constant will result in an increased dielectric loss. The low-dielectric-constant AIN layer is introduced under the ScAlN layer to design the laminated composite structure. This can significantly reduce the energy loss caused by the dielectric effect, thereby reducing the $\varepsilon_{r}$ value. This heterogeneous composite system not only maintains the advantages of the high voltage response of ScAlN, but also effectively inhibits the dielectric loss of a single-component material through the material property complementarity mechanism (Figure 2b for the specific structural configuration).

From the perspective of material mechanics, although the introduction of the ScAlN material can improve the theoretical charge output through a piezoelectric enhancement effect, its material characteristics will reduce the elastic stiffness constant $c_{33}$, and experiments show that the actual charge gain is limited and accompanied by a significant dielectric loss. The innovative design of AlN/ScAlN composite films can achieve a significant inhibition of dielectric loss while maintaining the original charge generation level through interface characteristic regulation, which breaks through the performance bottleneck of traditional uniform ScAlN films.

## 3. Hydrophone Fabrication

**Figure 3:**
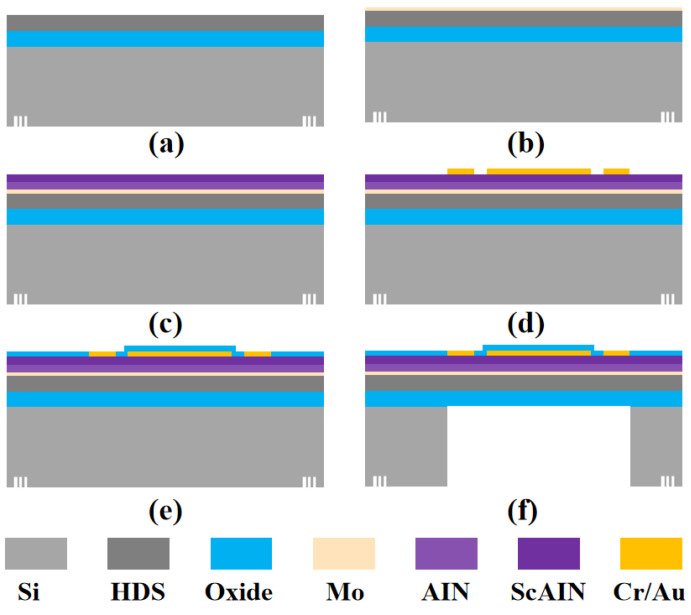
The production of hydrophone array process is as follows: (**a**) SOI wafer. (**b**) Deposition of a 0.15 μm Mo seed layer. (**c**) Deposition of 1 μm AlN/Sc_0.2_Al_0.8_N piezoelectric composite layer by magnetron sputtering. (**d**) Deposition and lithographic patterning of a 30/300 nm Cr/Au composite electrode layer to form the top electrode and PAD structure. (**e**) Deposition of a 0.3 μm oxide layer on top. (**f**) Etching of the oxide layer in the pad area to create an electrical contact window. DRIE from the backside to form the cavity and diaphragm structure of the hydrophone array.

The fabrication process of this device commences with a silicon-on-insulator (SOI) wafer, as illustrated in Figure 3a. The selected SOI wafer features a trilayer heterostructure; a 2 μm thick heavily doped silicon (HDS) top layer with resistivity below 0.005 Ω·cm, which effectively minimizes ohmic losses during carrier transport; a 2 μm buried oxide (BOX) intermediate layer; and a standard silicon substrate 500 μm thick.

The manufacturing sequence initiates with the deposition of a 0.15 μm molybdenum (Mo) seed layer via reactive magnetron sputtering on the SOI surface by an MSP-3200 sputtering system (Figure 3b). This metallic layer serves as an optimal template for subsequent piezoelectric film epitaxial growth due to its compatible lattice constant with aluminum nitride (AlN). A gradient deposition technique (Figure 3c) is employed for the piezoelectric functional layer, initially depositing a 500 nm AlN layer through physical vapor deposition using high-purity Al targets, followed by co-sputtering with Sc/Al dual targets to achieve a 20% Sc-containing ScAlN (Sc_0.2_Al_0.8_N) layer, ultimately forming a 1 μm thick AlN/ScAlN composite piezoelectric sensing structure by the Beneq TFS 200 coating system. Subsequent fabrication involves magnetron sputtering of Cr/Au (30/300 nm) bilayer electrodes, patterned through photolithography by an EVG610 lithography machine and using AE4 equipment argon ion beam etching (IBE) to define interdigital electrodes and bonding pads (Figure 3d). To enhance device reliability, a 300 nm SiO_2_ passivation layer is deposited via plasma-enhanced chemical vapor deposition (PECVD) by SI500D equipment (Figure 3e), with bonding area openings created through an RIE-10NR plasma etching machine. Final processing includes mechanical polishing to reduce substrate thickness to 345 μm, followed by back cavity formation using deep reactive ion etching (DRIE) by the Omega LPX Dsi system (SPTS Technologies Ltd., Newport, UK) (Figure 3f).

**Figure 4:**
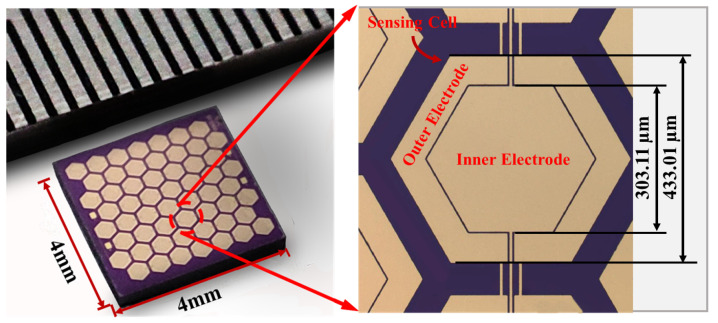
Optical image of hydrophone array and enlarged image of intermediate region.

As shown in Figure 4, optical microscopy images demonstrate the 4 mm × 4 mm piezoelectric composite membrane hydrophone array and detailed structural features of its central region. The micrographs reveal well-aligned, structurally intact hydrophone units. The PMUTs with a biological honeycomb structure achieve a fill factor of 81% compared to 64% with a regular arrangement of circular sensing cells, giving them higher sound pressure sensitivity. Compared to square diaphragms, the honeycomb sensor diaphragm has a uniform stress distribution and better stability.

**Figure 5:**
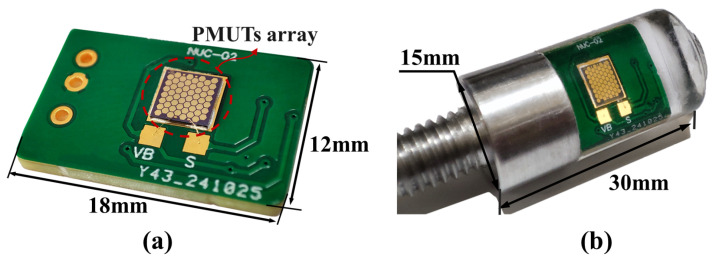
Integration and packaging of MEMS hydrophone system. (**a**) Electrically interconnect the MEMS acoustic sensing array to the printed circuit board using lead bonding. (**b**) Protective encapsulation of the integrated sensing unit by pouring PDMS.

**Figure 6:**
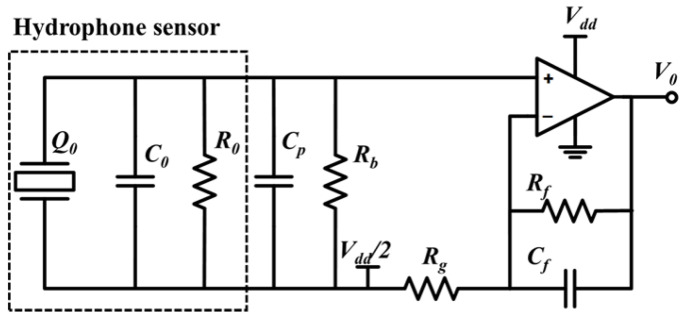
Schematic diagram of a voltage-mode amplification circuit.

Figure 5a shows the architecture of a miniaturized sensing system based on a piezoelectric composite membrane hydrophone. The PCB interconnecting system built via lead bonding technology effectively reduces the transmission noise and enables the pre-amplifier module to achieve a compact package of 1.8 cm × 1.2 cm × 0.16 cm. Acoustic impedance matching PDMS material was used for acoustic packaging in the experiment (Figure 5b), Using a two-stage compound in-phase amplifier circuit to achieve charge signal enhancement, as shown in Figure 6. The circuit design achieves 40 dB amplification through gain optimization and maintains stable gain characteristics in the frequency domain of 10–200 kHz.

As shown in Figure 5b, the PDMS is packaged in a cylindrical package whose thickest thickness is 6.3 mm. PDMS exhibits broadband characteristics in MEMS transducers because of its low elastic modulus (3 MPa) and acoustic impedance (1.5 MRayl), which closely match that of water. The PCB packaging scheme was required for test verification. This technical solution innovatively solves the problem of miniaturization and broadband response compatibility of underwater acoustic sensing system, and its broadband characteristics show significant application value in the field of underwater target feature analysis, providing a new technical paradigm for the development of scalable underwater acoustic detection systems.

## 4. Performance Characterization

The performance test and characterization of piezoelectric composite film hydrophone is a key step to verify its reliability and application effectiveness. Through the comprehensive evaluation of the mechanical, electrical and acoustic characteristics of the hydrophone, the performance level, reliability and effectiveness of the hydrophone can be accurately determined, and the optimization basis for underwater detection, acoustic monitoring and other applications can be provided.

### 4.1. Morphology and Crystal Orientation Testing

**Figure 7:**
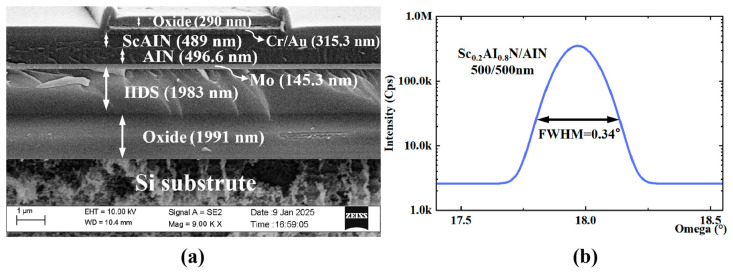
(**a**) SEM images of piezoelectric diaphragm sputtered with ScAlN/AIN films. (**b**) FWHM value of 0.34°. XRD rocking curve of piezoelectric composite films.

The reported piezoelectric composite film hydrophone was constructed on a silicon-on-insulator (SOl) platform, featuring a core structure incorporating an innovative AlN/Sc_0.2_Al_0.8_N composite piezoelectric sensing layer. This piezoelectric stack comprises 500 nm thick Sc_0.2_Al_0.8_N and AlN layers, with molybdenum (Mo) serving as the bottom electrode and a chromium/gold (Cr/Au) bilayer as the top electrode. The piezoelectric architecture is embedded between a 0.3 μm oxide surface layer and a dual-layer 2 μm dielectric stack containing an HDS layer and buried oxide layer, suspended above a 350 μm deep cavity-etched substrate. The top oxide protective layer effectively prevents electrode oxidation and interlayer shorting through isolation engineering. Figure 7a presents cross-sectional SEM characterization revealing detailed microstructural features of the sensing diaphragm. Experimental studies indicate that although the molybdenum substrate slightly increases surface roughness and acoustic transmission loss in AlN films, its high acoustic impedance substantially enhances the electromechanical coupling coefficient by improving interfacial acoustic wave reflection efficiency, thereby optimizing device performance.

In terms of performance optimization, the top electrode of the sensor unit adopts an internal and external partition design, and the electrode size ratio is controlled to 7:3. X-ray diffraction analysis Figure 7b shows that the composite piezoelectric film prepared by sputtering process exhibits a half-peak width of 0.34° on the (002) crystal surface, confirming its highly preferred orientation characteristics along the C-axis, which is significantly correlated with the piezoelectric properties of the material.

### 4.2. Vibration Testing

**Figure 8:**
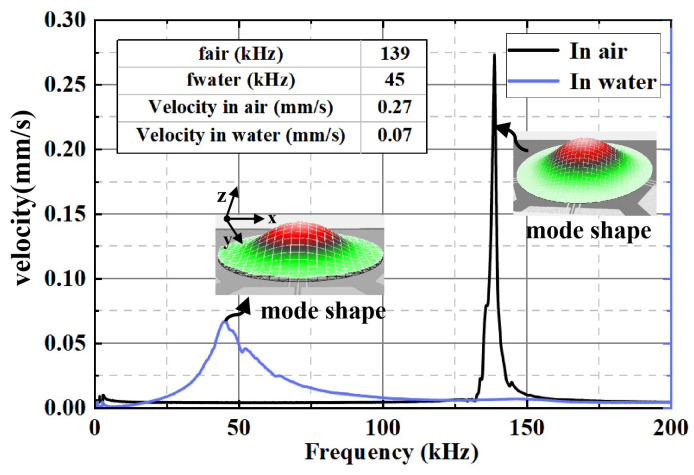
The frequency response of piezoelectric composite film hydrophone array in air and water was measured by Polytec MSA-600 LDV.

The vibration characteristics of the piezoelectric composite film hydrophone in air and water were evaluated using a Polytec laser Doppler vibrometer (MSA-600). During the frequency response measurement, the average amplitude of the sensing diaphragm within the hydrophone’s sensing unit was directly measured by the laser Doppler vibrometer (LDV) to characterize its frequency response features, with the relevant experimental results presented in Figure 8. Through precise measurements, the resonant frequencies of the hydrophone were accurately determined: 139 kHz in air and 45 kHz in water. Due to process variations in chip manufacturing during actual testing, the simulated material properties are more ideal compared to real samples, and there are uncontrollable factors that lead to discrepancies between the simulated resonant frequency and the actual measured results.

Subsequently, under the crucial condition of the resonant frequency, the surface vibration velocity of the hydrophone’s sensitive diaphragm in both air and water was tested.When a driving voltage of 0.1 Vpp (peak-to-peak) was applied in air, the velocity of the sensitive diaphragm could reach 0.27 mm/s. In contrast, when a driving voltage of 5 Vpp (peak-to-peak) was applied in water, its velocity was 0.07 mm/s.

### 4.3. Sound Pressure Sensitivity Testing

In the performance evaluation of packaged piezoelectric composite membrane hydrophones, the sound pressure sensitivity is a key index. In order to measure the sound pressure sensitivity accurately, the 3D Vector Standing Wave Tube Hydrophone Calibration System was used in this experiment.

The whole test process was carried out at room temperature, which provides a reliable environmental reference for the test results. The sound pressure sensitivity is defined as the voltage (V) applied to the phone in unit of sound pressure (μPa), expressed in dB re: 1 V/μPa. The calculation formula is as follows:

(10) $$S_{P}=20\mathrm{lg}\left(\frac{V_{out}}{P_{in}}\right)$$

where Vout is the output voltage and Pin is the input sound pressure (μPa). The sensitivity measured at 1 kHz is = −162.9 dB. The receiving sensitivity is defined as the electrical output efficiency of the hydrophone when the acoustic wave is incident, which is directly related to the sound pressure sensitivity, but the circuit gain must be considered. In this work, the equivalent circuit model is used to calculate the following:

(11) $$S_{RX}=\frac{G\cdot A\cdot FA}{\eta }\frac{3\eta^{2}Z_{ele}}{Z_{tot}+\eta^{2}Z_{ele}}$$

where G represents the voltage amplification factor of the pre-amplifier circuit, is the electromechanical coupling factor, Zele corresponds to the electrical impedance of the hydrophone element, Ztot represents the equivalent impedance under coupled mechano-acoustic field conditions, A represents the effective surface area of the hydrophone element, and FA represents the spatial efficiency factor of the sensor geometry, respectively. The receiving sensitivity is the value based on the theoretical analysis of the equivalent circuit model, and the sound pressure sensitivity is the value measured by the experimental device.

**Figure 9:**
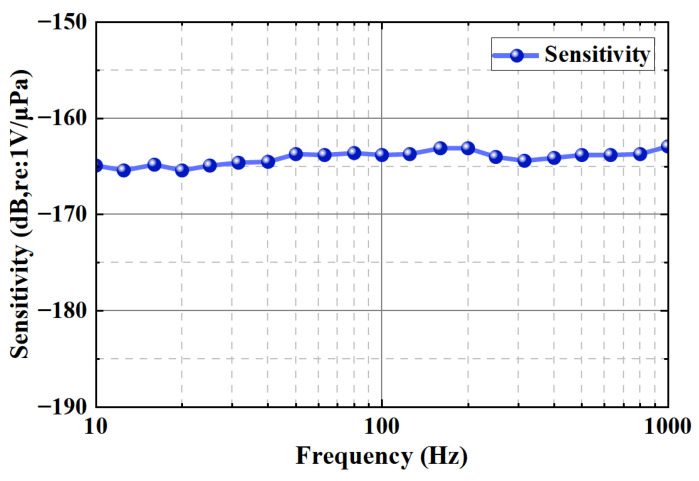
The piezoelectric composite film hydrophone has stable sound pressure sensitivity in the bandwidth range of 10 Hz to 1 kHz at room temperature.

Underwater acoustic detection indicators are typically used in the frequency range of 10 Hz to 1000 Hz. In the test process, the piezoelectric standard hydrophone provides a stable and suitable excitation signal for the hydrophone to be tested in the test frequency band. At the same time, the hydrophone to be tested receives and records the acoustic signal emitted by the standard hydrophone. The results show that the hydrophone has a consistent flat response in the bandwidth range. Among them, at room temperature, the sound pressure sensitivity is −163.9 ± 1 dB (re: 1 V/μPa) in the bandwidth range of 10 Hz to 1 kHz, and the receiving sensitivity is −162.9 dB in the range of 1 kHz (Figure 9). Experimental results demonstrate the hydrophone’s consistent sensitivity and frequency response characteristics within the tested bandwidth. Compared with similar hydrophones in the industry, the performance of the hydrophone is outstanding and in a leading position, which provides an important basis for performance evaluation in practical applications.

### 4.4. Directivity Testing

Directivity is a key index to evaluate the acoustic performance of hydrophones, which directly reflects the difference in spatial acoustic response characteristics. In this study, a directional characterization experiment was carried out for piezoelectric composite membrane hydrophone. A 1 kHz sinusoidal signal was emitted by a low-frequency standard sound source, and the 0–360° omnidirectional scanning was achieved by using a stepper motor to drive the measured device which is produced by Hangzhou Maihuang Technology Co., Ltd. (Hangzhou, China).

**Figure 10:**
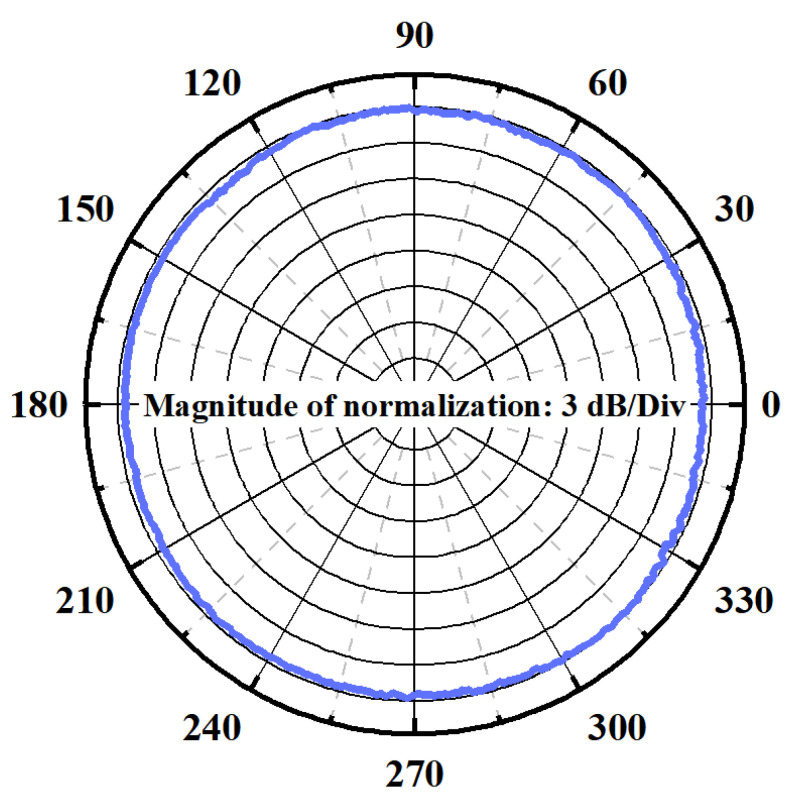
Directivity of piezoelectric composite film hydrophone measurement at 1 kHz.

The experimental data as shown in Figure 10 show that the hydrophone presents a fluctuation amplitude of ±0.5 dB of the polar coordinate pattern at the reference frequency, showing excellent omnidirectional sensitivity consistency. This uniform spatial response effectively overcomes directional selective interference in complex underwater sound fields, provides technical support for obtaining high-fidelity sound pressure signals, and significantly improves the environmental adaptability and measurement reliability of underwater detection systems.

### 4.5. Equivalent Noise Density Testing

Equivalent noise density (END) is a key parameter to measure the detection limit of a hydrophone, characterizing the lowest recognizable pressure level, which is determined by the inherent noise of the device. The lower the END, the stronger the hydrophone’s own noise suppression ability, and the more significant the advantage in weak signal detection. For example, in the study of deep sea bioacoustics, it is easy to drown the microacoustic signals of marine organisms with a high END, and a low END device can effectively extract such weak acoustic features.

In this work, the equivalent noise density is characterized by measuring the noise density characteristics of the hydrophone system. Noise density testing requires a hydrophone and its pre-amplifier circuit output to be connected to a spectrum analyzer (35670A) in a quiet environment. When the hydrophone is working properly, the data of the spectrum analyzer can be recorded. By bringing the measured data into the formula, the equivalent noise density of the hydrophone is obtained, which can be described as

(12) $$ND_{eq}=\frac{ND}{S}$$

where ND_*eq*_ is the noise density and S is the measured sound pressure sensitivity of the hydrophone sensor, respectively.

**Figure 11:**
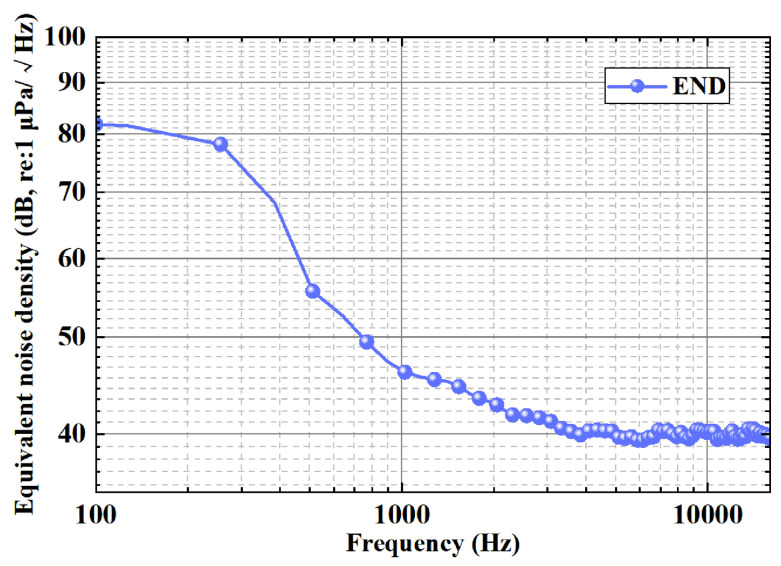
Frequency response of the END for the reported piezoelectric composite film hydrophone.

The results are shown in Figure 11. The END of the piezoelectric composite membrane hydrophone at 1 kHz is as low as 46.1 dB (re: 1 μPa/$\sqrt{\mathrm{Hz}}$). The END is negatively correlated with the sound pressure sensitivity, and the low END corresponds to the high sensitivity, indicating its strong response ability to the sound signal. High sensitivity can enhance the ability of remote weak signal acquisition and reduce the communication bit error rate.

Table 2 demonstrates that the AlN/Sc_0.2_Al_0.8_N-based piezoelectric composite film hydrophone array has achieved a commercially advanced level in key performance metrics such as sensitivity, directivity, and equivalent noise density. The piezoelectric sensing layer exhibits a highly preferred C-axis orientation and superior crystal quality. Research findings indicate that this sensor overcomes the technical limitations of traditional material systems, with its overall performance rivaling that of existing commercial hydrophones. Moreover, it offers distinct advantages in miniaturization and integration, presenting an innovative solution for next-generation underwater acoustic sensing technology and showcasing significant commercial application potential.

## 5. Conclusions

In this work, a novel underwater acoustic sensor based on AlN/Sc_0.2_Al_0.8_N piezoelectric heterothin films has been developed. Through the design of a non-uniform composite sensitive layer, the medium loss and defect density are reduced while retaining the high piezoelectric properties of scandium-doped aluminum nitride, and the tradeoff between the performance and reliability of traditional devices is overcome. The 7 × 8 microarray (total size 4 mm × 4 mm) prepared based on an SOI platform combined with PDMS package achieves 45 kHz underwater operating frequency. X-ray diffraction analysis showed that the films have excellent crystal quality (FWHM of 0.34°). Acoustic test results show that the transducer exhibits omnidirectionality at 1 kHz frequency (with a deviation of 0.5 dB), an acoustic pressure sensitivity of −162.9 dB (re: 1 V/μPa), and an equivalent noise density as low as 46.1 dB (re: 1 μPa/*√*Hz).

The performance of the device reaches the commercial high-end standard, and its silicon-based compatibility provides a new solution for the miniaturization of underwater acoustic sensing. Compared with the existing technologies, this study achieves the synergistic optimization of high-voltage electrical response and low dielectric loss through the design of heterogeneous composite films, and also successfully prepares 7 × 8 microarrays (with a total size of 4 mm × 4 mm) based on the SOI platform and the PDMS packaging technology, which provides the feasibility of miniaturization and mass production of underwater acoustic sensors. In addition, the omnidirectionality and low-noise characteristics of the sensor allow it to have a wide range of potential applications in complex underwater environments, such as marine resource exploration, underwater communication and military monitoring.

## Figures and Tables

**Table 1:** Parameters of the AlN/Sc_0.2_Al_0.8_N-based piezoelectric composite film hydrophone array.

Hydrophone Layer	Material	Size (μm)	Thickness (μm)
Top oxide layer	SiO_2_	-	0.3
Top electrode layer	Au	-	0.3
	Cr	-	0.03
Piezoelectric layer	Sc_0.2_Al_0.8_N	-	0.5
	AlN	-	0.5
Seed layer	Mo	-	0.15
HDS layer	high-doped Si	-	2
Bottom oxide layer	SiO_2_	-	2
Substrate	Si	-	345
Cavity	Void	500	345

**Table 2:** Performance comparison of the developed piezoelectric composite film hydrophone with commercially available advanced hydrophones.

Hydrophone	Cetacean CR2 [16]	Reson TC4047 [17]	Ref. [3]	Ref. [22]	This Work
Technology	Piezoceramic	Piezoceramic	MEMS (AIN)	MEMS (ScAIN)	MEMS (AIN/ScAIN)
Chip Size	N.A.	N.A.	3.5 mm × 3.5 mm	4 mm × 4 mm	4 mm × 4 mm
Sensivity (dB, re: 1 V/μPa)	−214 ± 3	−191 ± 3	−180 ± 1	−172 at 1 kHz	−162.9 ± 1
Beam Pattern	N.A.	N.A.	N.A.	±0.5 dB at 1 kHz	±0.5 dB at 1 kHz
END (dB, re: 1 μPa/*√*Hz)	68 at 1 kHz	51 at 1 kHz	60 at 1 kHz	N.A.	46.1 at 1 kHz

## Data Availability

The data are available upon request from the authors.
